# Two-Dimensional Non-Carbon Materials-Based Electrochemical Printed Sensors: An Updated Review

**DOI:** 10.3390/s22239358

**Published:** 2022-12-01

**Authors:** Shaili Falina, Khairu Anuar, Saiful Arifin Shafiee, Joon Ching Juan, Asrulnizam Abd Manaf, Hiroshi Kawarada, Mohd Syamsul

**Affiliations:** 1 Collaborative Microelectronic Design Excellence Center (CEDEC), Universiti Sains Malaysia, Sains@USM, Bayan Lepas 11900, Pulau Pinang, Malaysia; 2 Faculty of Science and Engineering, Waseda University, Tokyo 169-8555, Japan; 3 Department of Chemistry, Kulliyyah of Science, International Islamic University Malaysia, Bandar Indera Mahkota, Kuantan 25200, Pahang, Malaysia; 4 Nanotechnology & Catalyst Research Centre (NANOCAT), Institute of Postgraduate Studies, University Malaya, Kuala Lumpur 50603, Malaysia; 5 The Kagami Memorial Laboratory for Materials Science and Technology, Waseda University, 2-8-26 Nishiwaseda, Shinjuku, Tokyo 169-0051, Japan; 6 Institute of Nano Optoelectronics Research and Technology (INOR), Universiti Sains Malaysia, Sains@USM, Bayan Lepas 11900, Pulau Pinang, Malaysia

**Keywords:** 2D materials, non-carbon, electrochemical, screen printed electrode, sensors, TMDCs, MXenes, hexagonal boron-nitride

## Abstract

Recently, there has been increasing interest in electrochemical printed sensors for a wide range of applications such as biomedical, pharmaceutical, food safety, and environmental fields. A major challenge is to obtain selective, sensitive, and reliable sensing platforms that can meet the stringent performance requirements of these application areas. Two-dimensional (2D) nanomaterials advances have accelerated the performance of electrochemical sensors towards more practical approaches. This review discusses the recent development of electrochemical printed sensors, with emphasis on the integration of non-carbon 2D materials as sensing platforms. A brief introduction to printed electrochemical sensors and electrochemical technique analysis are presented in the first section of this review. Subsequently, sensor surface functionalization and modification techniques including drop-casting, electrodeposition, and printing of functional ink are discussed. In the next section, we review recent insights into novel fabrication methodologies, electrochemical techniques, and sensors’ performances of the most used transition metal dichalcogenides materials (such as MoS_2_, MoSe_2_, and WS_2_), MXenes, and hexagonal boron-nitride (hBN). Finally, the challenges that are faced by electrochemical printed sensors are highlighted in the conclusion. This review is not only useful to provide insights for researchers that are currently working in the related area, but also instructive to the ones new to this field.

## 1. Introduction

Electrochemical sensors have been widely used to detect various analytes of interest and display high sensitivity and selectivity, especially when combined with the use of two-dimensional nanomaterials with highly catalytic properties. The advancement in miniaturization and microfabrication of electrochemical sensors has led to renewed interest in the development of disposable electrodes as an analytical power tool. The application of disposable electrodes as point-of-care diagnostics and in situ analytical monitoring devices in biomedical, pharmaceutical [1], industrial [2], food safety [3], and environmental fields [4] has been the major focus of research in this field. Printing electrodes is currently the most popular method for fabricating disposable sensors. It is a fast and rapid method to produce sensors in mass volume. Additionally, it is also low-cost, as its fabrication calls for the use of different cheap substrates such as ceramics, paper, or plastics [5,6,7]. The electrodes can be printed with carbon or graphite ink, which is also economical compared to the fabrication of traditional electrochemical sensors.

Printed electronics have emerged as a current groundbreaking technology in electrochemical sensor fabrication, mainly due to advances in printable metallic, insulating, and semiconductor materials as well as mature printing techniques. In the early stages, printed electronics were focused on the display and lighting industries [8,9]. However, as time goes on, the printed electronic industries have broadened towards electronic devices [10,11,12], sensors [13,14,15], and circuits [16,17,18]. To date, various printing techniques have been introduced and developed, including screen-printing, off-set printing, gravure printing, spray coating, ink-jet printing, and many other hybrid printing techniques. Among these techniques, screen-printing and inkjet-printing are the popular choices to fabricate printed sensors. Standard sensor fabrication employs blankets of material deposition, photolithography, and etching procedures that are not only complicated, but also expensive and wasteful. Contrastingly, for the sensor that is fabricated through the printing method, only minimal and selective material deposition is required. Cost efficiency, high volume, and roll-to-roll manufacturing are major strengths of printed sensors. Beyond that, it also allows for the capability of printing sensor devices on soft, flexible, thin, and unconventional substrates, which enables wearable sensor applications. Healthcare is currently the biggest application field for printed sensors. To date, printed sensors have been widely studied as wearable and point-of-care (PoC) diagnostic tools.

**Figure 1:**
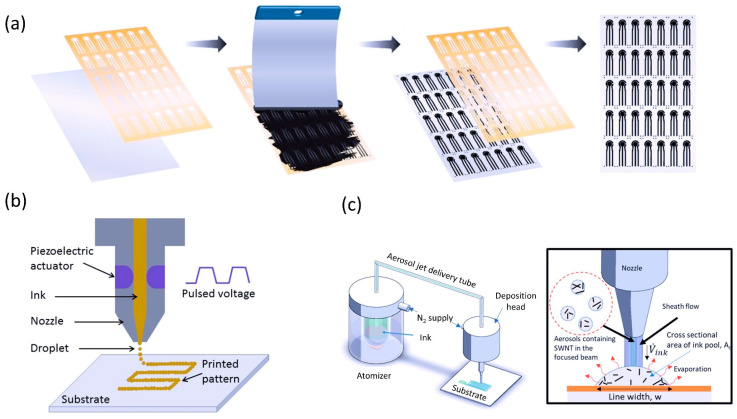
Printed sensor fabrication techniques (**a**) Screen-printing. Reproduced with permission from [45]. Copyright 2018 Plos ONE. (**b**) Ink-jet printing. Reproduced with permission from [46]. Copyright 2018 IOP Publishing All rights reserved. (**c**) Aerosol jet printing and its zoomed-in schematic of aerosol jet deposition. Reprinted (adapted) with permission from [47]. Copyright 2019 American Chemical Society.

Generally, printable electrochemical sensors can be categorized into two groups: (1) contact printing, which includes gravure, flexographic, off-set printing, and screen-printing, and (2) non-contact printing comprising inkjet printing, aerosol jet printing, laser-induced forward transfer (LIFT), micro and nano-pen printing [19,20,21,22]. The contact printing generally employs a mask-based printing technique in which a particular pattern is inked onto a substrate through a mask. In this technique, the substrate and mask are in physical contact. On the other hand, non-contact printing is a maskless printing technique in which the ink is dispensed through openings or nozzles. The sensor structure is defined by moving the stage in a pre-programmed pattern. In contrast to contact printing, non-contact printing has benefits in terms of reduced material waste, ameliorated resolution, enabling miniaturization, and complex pattern printing. Figure 1 below illustrates the printing techniques that are commonly used to fabricate electrochemical sensors.

At present, a vast body of literature has been published on 2D materials for sensing applications. Graphene and the other carbon materials (graphene oxide, graphene reduced oxides, carbon nanotubes, etc.) have attracted significant attention from academic and commercial researchers owing to their exceptional properties, such as biocompatibility, adjustable surface properties, fast electron transfer capability, and large surface area [23,24,25,26]. Nevertheless, the families of 2D non-carbon materials have also shown rapid advancement along with the 2D carbon materials. Most of the non-carbon materials share similar properties with those of carbon materials. However, the non-carbon materials certainly have advantages over the carbon materials in some areas. For example, MXenes and MoS_2_ have been in the spotlight for electrochemical sensing applications due to their tunable bandgap property [27,28], which help enhance sensing performance by reducing the signal-to-noise ratio. The non-carbon materials also offer an extensive range of materials compared to carbon materials. Up to date, about 40 different TMDC compounds exist with astonishing properties and the possibility of interconversion to various nano-dimensionalities such as 0-dimension (0D), 1-dimension (1D), and 2-dimension (2D) structures [29]. Although hexagonal boron nitride (hBN) materials are not very popular as sensing materials due to their insulation property, they are a very useful material for reinforcement of polymeric films [30]. While these non-carbon materials demonstrate that they exceed the merits of the carbon families, several drawbacks also have been identified. MXenes have poor mechanical property, easy restacking (aggregation), and relatively small lateral size, as well as poor stability in oxygen atmosphere, thereby restricting its application for highly durable and flexible wearable sensors [31]. When compared to graphene, TMDC materials have relatively low carrier mobility (200 cm^2^ V^−1^ s^−1^ in the case of MoS_2_, in contrast to graphene, where its carrier mobility can be up to 200,000 cm^2^ V^−1^ s^−1^) [32]. In principle, the hBN surface is a molecularly smooth basal plane without any functionalities. To be able to operate as a sensing material, surface modification is usually essential in the sensor fabrication step. It has been reported that modification and functionalization on the hBN surface was quite a challenge, attributed to its dense structure [33].

This review aims to provide an overview of recent studies in the development of non-carbon two-dimensional (2D) materials on printed electrochemical sensors over the past five years (from 2018 to present). During this period, many review articles have been published in the field of printed sensors and 2D materials. It may, however, be noted that most of the review articles were focused on the advancements in 2D materials on electrochemical sensors in general [34,35,36,37,38,39,40]. Herein, we narrow down the scope of our studies to advance electrochemical printed sensor integration with non-carbon 2D materials as sensing platforms. We present our materials in three main sections covering transitional metal-dichalcogenides (TMDCs), MXenes, and hexagonal boron-nitrides (hBN). Particular emphasis has been given to the most common TMDCs materials, including molybdenum disulfides (MoS_2_), molybdenum diselenides (MoSe_2_), and tungsten disulfides (WS_2_). We cover innovative breakthroughs and applications of these 2D materials in the development of next generation printed electrochemical sensors. Although it should be borne in mind that this review is restricted to printed electrochemical sensors for biomolecule detection (biosensor), pharmaceutical, food safety, and environmental monitoring. Humidity and gas printed electrochemical sensors are excluded from this review as they have recently been comprehensively reviewed elsewhere [41,42,43,44]. Beyond that, this review also presents the principle and operation of printed sensors, electrochemical technique analysis, as well as surface modification and functionalization specifically on printed electrochemical sensors in the first part of the article. This review is intended to give an update on recent advances, key developments, and applications. The authors sincerely apologize for any possible oversight of other key papers in this field.

## 2. Printed Electrochemical Sensors and Electrochemical Technique Analysis

**Figure 2:**
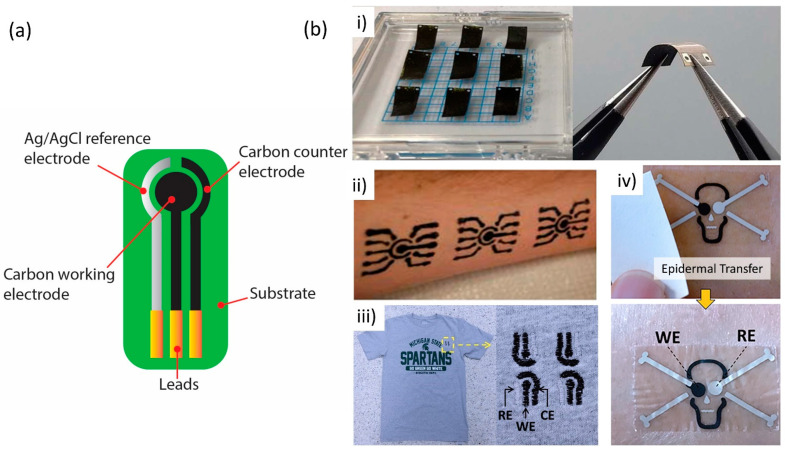
(**a**) A scheme of construction for a printed electrochemical sensor. The printed electrochemical sensors consist of three electrode systems, the working electrode, the counter electrode, and the reference electrode. (**b**) Printed sensor electrodes on various types of substrates, (**b-i**) printed electrochemical pH sensor on a flexible thin film. Reproduced/Adapted from Ref. [55] with permission from The Royal Society of Chemistry, (**b-ii**) sensor arrays printed on the skin epidermis. Reproduced/Adapted from Ref. [56] with permission from The Royal Society of Chemistry, (**b-iii**) printed sensors on textile and its zoomed-in image. Reproduced/Adapted from Ref. [57] with permission from The Royal Society of Chemistry, and (**b-iv**) printed chemical sensor on tattoo transfer paper. Reprinted with permission from [58]. Copyright 2018 Elsevier.

The fabrication of printed electrochemical sensors frequently follows similar ideas to those of standard electrochemical electrodes in terms of structure. Generally, it is fabricated as a three-electrode system, which encompasses a working electrode (WE), a reference electrode (RE), and a counter electrode (CE). The electrode components are constructed by printing different inks in successive layers to create a final product. The printed electrodes often require curing procedures before use to anneal the layer of ink together with the substrate underneath for better stability [48]. The WE and CE are usually printed with the same conductive ink material, while the RE is usually printed with silver/silver chloride (Ag/AgCl) conductive ink, to replicate the reference Ag/AgCl electrode in the standard electrochemical set-up. The electrochemical sensor performance corresponds to a few factors, including structure, loading mass, specific areas, mechanical property, and ink formulation of the printable electrodes. Therefore, it is important to choose high performance electrode materials and ink formulations with exceptional rheological properties. For WE and CE, carbon and graphite inks are two common inks that have widely been used as printable electrode sensors owing to their electrically conductive properties and capability to electrocatalyze many oxidation/reduction processes [49,50]. A number of studies have employed silver tracks underneath the carbon or graphite printed electrodes to improve the electrode’s conductivity [51,52,53]. Thanks to their high conductivity, gold, silver, and platinum material inks also make good printable electrodes for sensor applications. However, due to their higher cost, these inks are less popular choices compared to carbon and graphite ink. As regards gold ink, the generation of self-assembled monolayers (SAM) on gold surface through a strong Au-S bond has attracted many researchers to employ gold ink in screen-printed electrode (SPE) fabrication [54]. Besides electrode ink material, another important component in printed sensor fabrication is the substrate designer’s intended application purposes. For example, for health monitoring applications, the sensor electrode is usually printed on tattoo transfer paper or directly stamped on the electrodes on the skin’s epidermis. Some of the health monitoring sensors are printed on textiles. These sensors are customarily known as wearable sensors. For other applications, sensor electrodes can also be printed on thin-film plastic. Polyethylene terephthalate (PET), polyethylene naphthalate (PEN), and polyimide (PI) are the thin-film plastics that are frequently used for printing electrode sensors. However, it should be noted that these substrates must withstand heat transfer during the annealing process. Figure 2a,b show the three-electrode system of printed electrochemical sensors and printed electrochemical sensors on various types of substrates, respectively.

Next, we outline electrochemical sensing techniques such as amperometric, impedimetric, potentiometric, and voltammetric sensing. A detailed discussion of these electrochemical techniques has been excellently reviewed by Sardini et al. [59]. Nevertheless, we would like to lay out a short overview and conception to readers about analysis techniques that are usually used for electrochemical printed sensors.

(1) Amperometric—Amperometric techniques measure the resultant current from oxidation and reduction activities of an electroactive species, exchanging electrons with WE conductive surface, where this current is measured at a constant voltage. The advantage of this particular analysis technique is that it allows for highly sensitive and rapid analysis. Despite its convenience, this analysis technique tends to suffer from cross-sensitivity, interferences with buffer composition, and the effects of surrounding solution [60]. Chronoamperometry is the advanced version of the amperometry technique. A steady current is measured as a function of time, when a controlled potential technique with a stepwise profile is applied at the working electrode. In this case, the reduction and expansion of diffusion layer at the electrode surface cause the alternation in current. This type of analysis can be used to measure current–time dependence for the electroactive species activity occurring at the WE.
(2) Impedimetric—Electrochemical impedance spectroscopy (EIS) has received significant interest from researchers for sensor analysis, particularly in biosensors due to its advantage where only a low voltage is required, which does not destroy or interrupt the bio-recognition layers on WE [61]. EIS measures the direct correlation of impedance changes with changes of target analyte concentration. Concisely, the biomolecules bound onto the printed electrode act as an insulator. A small alternate voltage is applied to WE and CE with a constant amplitude (typically from 5 mV to 10 mV) and a defined frequency range (usually from 100 kHz to 1 mHz), resulting in an alternate current response. EIS data are commonly accompanied by Nyquist plot (Z_imag_ against Z_real_). The data collected with EIS can be modeled with appriopriate equivalent electrical circuit according to the electrochemical events occuring in the sample. The parameter’s values are adjusted until the mathematical function is comparable to the experimental data within a certain margin of error.
(3) Potentiometric—Potentiometric analysis technique is performed at zero-current conditions to measure the accumulation of charge potential at WE compared to RE in an electrochemical activity. The potential of WE depends on the concentration of the analyte (accumulation of charges will proportionally decrease or increase the potential of WE depending on the electrode surface), while the RE is essential to provide a potential reference.
(4) Voltammetric—Cyclic voltammetry (CV) is an overwhelmingly popular technique to obtain information of redox potential and electrochemical rates of the analyte. In this technique, a series of voltages are applied to the sensor electrode, and the resulting current is measured. The CV technique is often carried out in a partial cycle, one full cycle, or a series of cycles to study redox processes and monitor reaction intermediates as well as reaction product stability. Aside from CV, differential pulse voltammetry (DPV) also falls in the class of voltammetric techniques. Unlike CV, DPV measures the current immediately before each potential change. Therefore, the DPV graph represents current variation as a function of potential. Another major voltammetric technique that is often found in literature is square-wave voltammetry (SWV). SWV is an analysis technique that superimposes a high amplitude, high frequency square-wave signal onto a fast staircase waveform as the input, where the output is response current measured in forward pulse and reverse pulse. SWV is usually employed to study the mechanism, kinetics, and thermodynamics of a chemical reaction. One distinct advantage of SWV analysis is that it offers a very sensitive direct analysis down to parts per trillion (ppt) level [62].
(5) Chronocoulometry—Chronocoulometry analysis is performed to quantify cumulative charge (Q) that passes through electrochemistry electrode as a function of time. This analysis technique is established based on Faraday’s first and second laws of electroanalysis [63], where (1) the amount of deposited material on the electrode surface (during electrolysis) is directly proportionate to the quantity of electricity (Q) that passes through the electrolyte, and (2) the mass of ions deposited on the electrode are equivalent mass with the subtance electrolyzed. When a single or double pulse of potential is applied to the electrochemistry electrode, the resulting charge accumulation curve vs. time response can be obtained. Chronocoulometry has the same input potential as the chronoamperometry analysis; however, they provide different output results. Chronocoulometry output is the charge (Q), whereas chronoamperometry output is the current (*I*).

## 3. Surface Modification and Functionalization of Printed Sensors

**Figure 3:**
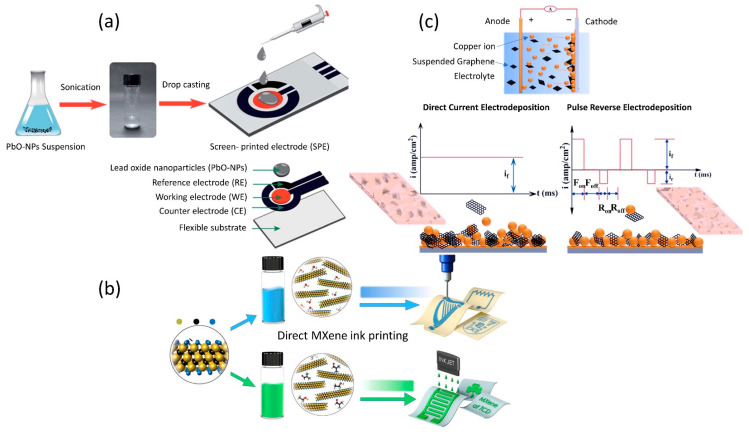
Surface modification or functionalization techniques of printed electrochemical sensors with nanomaterials. (**a**) Drop-casting technique. Reproduced/Adapted from Ref. [89] with permission from The Royal Society of Chemistry. (**b**) Ink-mixing and printing technique. Reprinted with permission from [90]. Copyright 2014 Springer Nature. Authors licensed under a Creative Commons Attribution (CC BY) license. (**c**) Electrodeposition technique. Reprinted with permission from [91]. Copyright 2019 Springer Nature. Authors licensed under a Creative Commons Attribution (CC BY) license.

To function as a sensor, the printed working electrode (sensing area) often requires modification with a layer of nanomaterials to enhance the electrochemical response towards the anticipated target analyte. Some of the sensors, specifically printed biosensors, call for functionalization of their WE with bio-species such as antibodies, enzymes, aptamers, and DNA as their sensing probes to capture the desired target. The three most common strategies for integrating a modifier and the sensing probe onto the printed working electrode are the drop-casting technique, electrodeposition, and ink formulation for direct printing. It is vital to ensure that the modifier is firmly attached to the working electrode and will not be dissolved in the surrounding solution. The attached sensing elements on the electrode are the determining factor for the performance of a reliable sensor. Drop-casting is an overwhelmingly popular approach for the preparation of printed sensors’ electrodes. In this approach, a drop of liquid containing suspension of material of interest is drop-cast on the sensing electrode as in Figure 3a, ideally confined to the electrode without overspill outside the electrode surface area. Typically, for surface modification with nanomaterial using the drop-casting method, the electrode surface is left to dry before use. Recently, Kumar and colleagues wrote an excellent review of drop-casting technique reliability from an experimental perspective [64]. The review provides an in-depth understanding of drop-casting challenges and to what extent they can result in a reproducible surface. The authors discussed the main concern associated with the drop-casting technique, which is coffee ring and its related effect. Coffee ring effect is a ring-like pattern that generally occurs when the sessile droplet containing suspended particle is evaporated in ambient conditions. Deegan et al. [65] thoroughly observed and examined the liquid’s behavior upon drying, which he interpreted in respect of the coffee stain. According to Deegan, taking a spilled drop of coffee as an example, at first the coffee particles are dispersed over the entire drop and slowly become concentrated at the periphery, in contrast to the center of the stain. Notably, the coffee ring results in a disruption of uniform material particle distribution [66,67,68] and therefore generates a suboptimal electrochemical response. Because of uncontrollable particle distribution, a sensor that utilizes the drop-casting technique for surface modification is rather difficult to reproduce. Kumar et al. also highlighted the efforts that have been made to mitigate the coffee ring effect, such as (1) choosing super hydrophobic surfaces can help to suppress the coffee ring effect by inducing the inward circulatory flow of the solute particles, resulting in more homogeneous deposition, (2) utilizing electrowetting method, which helps make the deposited solute particles mobile and not static, (3) modifying the shape of solute particles from spherical to ellipsoidal. Ellipsoidal particles demonstrated stronger long-range inter-particle capillary attractions between the solute particles, thereby leading to more uniform distribution, (4) introducing surface acoustic waves (SAWs) into evaporating droplets. This strategy is essential to trap solute particles on the substrate rather than moving forward to the edge by applying a pressure gradient at the surface, and (5) last but not least, employing Marangoni flow to create a surface tension gradient over the liquid-air interfaces of the solute particle droplet. The Marangoni flow aids in forming a concentrated solute center after evaporation when it is applied inward to the solute particle droplet to overcome outward capillary flow.

The formulation of printable ink with functional materials for sensing applications is an extremely important revelation for printed sensors. It allows for direct sensor printing without adding an extra step of sensor surface modification as shown in Figure 3b. Furthermore, with this innovative ink formulation, the functional moieties on top of the sensing electrode can also be more homogenous, and the printed sensor has a higher chance for reproducibility. In principle, printable ink for sensors mainly comprises active materials, binders, additives, and solvents. The main components of the ink are active materials including carbon black materials [69], semiconductor nanomaterials [70], conductive materials [71], electroactive materials [72] and so forth. The sensing material, such as biocompatible materials (enzyme, DNA, aptamer), polymers, various gels, and metals, is incorporated into the active electrode materials to function as sensing probes to detect the anticipated target and increase the electrocatalytic activity of the sensor electrode. Diversiform printable materials have greatly broadened the horizons of printed sensors as analytical and diagnostic tools for both research and commercial purposes. There have been a few attempts to tailor ink composition with functional materials for sensing purposes. For example, enzyme-containing inks are used for enzyme-based electrode sensor direct printing. The primary concern with enzyme ink is that it has a low shelf life on account of enzymes in suspension tending to rapidly lose their catalytic activity. Another potential disadvantages of printing electrodes using the enzyme-containing ink are the absorption of non-specific binding (NSB) and stability loss during the printing process as a consequence to thermal and mechanical stress [73]. To solve these problems, considerable work has been done to promote better stability and catalytic activity of the enzyme in the ink admixture, for instance, by employing an additive, using “immobilized enzyme”, enzyme rigidification, and fine-tuning enzyme attachment [74,75,76]. Direct sensor printing with customized ink containing polymers has also been reported. Bardpho et al. customized homemade graphene-polyaniline (G-PANI) ink to modify screen-printed carbon electrodes (SPCEs) for chromatographic determination of antioxidants in teas [77]. The G-PANI layer was inkjet-printed on the working electrode of the SPCE. In this work, PANI material was used to not only increase the electron transfer kinetics of the sensor electrode when combined with graphene, but it also played a role in preventing graphene agglomeration and improving graphene distribution on the electrode surface. Teengam et al. used the same recipe as Bardpho et al. to prepare G-PANI ink for surface modifier of his electrochemical paper-based peptide nucleic acid biosensor for human papillomavirus [78]. This study employed three printing techniques: (1) wax printing to create a hydrophobic barrier on a paper substrate, (2) screen-printing to fabricate the three electrodes of the sensor system (WE, RE, and CE), and (3) inkjet printing to modify the working electrode with G-PANI. In another study, Kit-Anan et al. and colleagues synthesized PANI ink in the laboratory and used the ink to print over the carbon working electrode (WE) as a sensor modifier to determine ascorbic acid [79]. The modifier ink was solely PANI material, and it was printed using an ink-jet printer to achieve a homogenous PANI layer on the sensing surface (WE).

Electrodeposition of functional materials on sensor electrodes has been studied extensively in the past decades. To date, various methods of electrodeposition have been developed to integrate engineered nanomaterials directly onto sensor electrodes, including electrochemical, electrospinning, and electrospray. In this review, we would like to focus on electrochemical deposition as it is a cost-effective technique and a common method found in the literature to modify the sensing electrode of printed sensor compared to the other two electrodeposition methods. Nevertheless, the electrospinning and electrospray deposition techniques have been discussed in detail elsewhere [80,81]. Electrochemical deposition offers rapid synthesis time with the absence of oxidant and reductant [82]. Moreover, the modifier material is directly deposited on top of the working electrode (sensing area), and it provides better adhesion [83]. Electrochemical deposition is a facile deposition technique that can be done mostly at room temperature and does not require a high vacuum setup. Three electrodes (WE, RE, and CE) are dipped into a desired material solution, and sufficient excitation potential is applied to give an external force for ions in the solution to move and reduced at the working electrode. A schematic of an electrochemical deposition setup is shown in Figure 3c. Cyclic voltammetry, double-pulse deposition, and potential step are the frequently used techniques for electrochemical deposition reported in the literature [84]. In previous studies, Rodríguez-Sánchez et al. established that the deposition particle size can be controlled precisely by adjusting the current density or applied potential and electrolysis time [85]. Tu et al. fabricated a disposal biosensor using one-step electrochemically deposited reduced graphene oxide (OerGO) onto a SPCE [86]. This sensor was exploited for L-lactate detection, an early cancer biomarker. The authors reported a successful rapid detection of L-lactate using this OerGO/SPCE sensor with only a tiny amount of sample (10 μL) within 10 s. Hondred et al. designed a printed graphene sensor for the direct and rapid monitoring of triple-O linked phosphonate organophosphates (OPs) [87]. The graphene electrode was printed using Inkjet Maskless Lithography (IML) technique, followed by electrodeposition of platinum nanoparticles (PtNPs dia. ~25 nm) to improve its electrical conductivity before conjugation with phosphotriesterase (PTE) enzyme for probing purposes. It has been observed that the sensitivity of the sensor improves as the number of deposition pulses or cycles increases (0 to 75 cycles, corresponding to sensor sensitivity of 195 and 275 nA/μM, respectively). Another study demonstrated the electrodeposition of flower-like gold microstructures (FLGMs) on top of the SPCE working electrode in sensor fabrication for serpin A12 detection [88]. To achieve superior sensor performance, the FLGM’s deposition time was optimized to 150 s. Subsequently, serpin A12-specific thiolated aptamer was covalently on the FLGMs as a probe to detect serpin A12. The sensor exhibited good reproducibility, selectivity, and sensitivity, as well as stability.

## 4. Transition Metal Dichalcogenides (TMDCs)

In recent years, there has been a greater focus placed upon transitional metal dichalcogenides (TMDC) within the sensor literature owing to their unique properties for sensing applications, such as excellent stability, biocompatibility, adjustable performance, fast heterogeneous electron transfer, and high specific surface area [92,93,94]. Moreover, TMDC materials also have the advantages of photothermal properties, fluorescent properties, and high density of electronic states making them popular for bioimaging [95,96,97].

**Figure 4:**
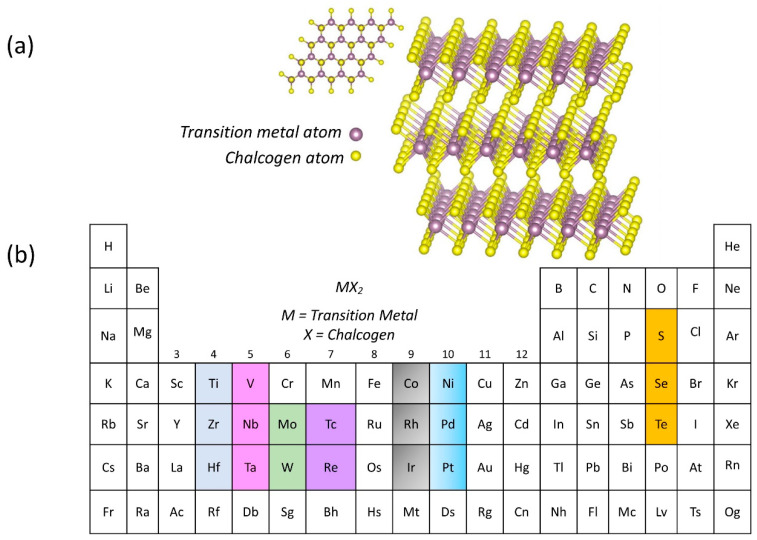
(**a**) General structure of TMDC, where the metal atom (purple) is sandwiched between two chalcogenides atoms (yellow). Reprinted with permission from [100]. Copyright 2021 Springer Nature. Authors licensed under a Creative Commons Attribution (CC BY) license. (**b**) There are more than 40 combinations of transition metal and chalcogen atoms that can form stable TMDC materials. The transition metals and the three chalcogens elements are highlighted in the periodic table.

The structure of TMDC materials is determined by one transition metal atom that is covalently linked with two hexagonal planes of chalcogen atoms in a sandwich fashion, which gives the material formulation of X-M-X, where M refers to transition metal atoms such as tungsten (W), molybdenum (Mo), titanium (Ti), and a few other metal atoms, while X represents chalcogen atoms such as sulfur (S), selenium (Se), and tellurium (Te). In the periodic table, Figure 4a,b exhibit the general structure of a single layer of TMDC material and TMDC elements (metal transition and chalcogen atoms), respectively. TMDC bulk consists of numerous layers of X-M-X stacked on top of each other and held together through Van der Waals force; therefore, the bonds between the adjacent layers are remarkably weak, although the chemical bond between atoms within each layer is strong and stable [98]. Interestingly, TMDC also possesses a wide range of electrical properties from insulator to superconductor, making it a promising material for an extensive range of applications. For example, HfS_2_ is an insulator, MoS_2_ is a semiconductor, WTe_2_ and TeS_2_ are semi metals, while NbS_2_ and VSe_2_ belong to the true metals group [99].

### 4.1. Molybdenum Disulfide (MoS_2_)

Among all the TMDC materials, molybdenum disulfide (MoS_2_) is the most popular material for sensing applications in the TMDC family, owing to its robustness. On top of that, high surface area and direct tunable bandgap makes MoS_2_ a promising candidate to extend the detection range with considerable responsivity [101]. In recent years, the direct printing method using nanomaterial inks to fabricate printable sensors has drawn more attention as this method offers time efficiency and is more suitable for sensor mass production. However, this method requires optimization at the ink development level, for example, at the viscosity recovery rate, flow behavior, and oscillatory modulus, to ensure the developed ink is adequate for the intended printer or printing process. Pavličková et al. [102] and her research group successfully fabricated a screen-printed MoS_2_ electrochemical sensor for dopamine sensing for the first time. Interestingly, the research group developed their own MoS_2_ ink before printing on the fluorine-doped tin oxide (FTO) substrate as sensor electrodes. In this work, they developed the ink with varying MoS_2_ particle size and volume to investigate the effect of these two parameters on dopamine sensing. The 6 μm and 90 nm MoS_2_ particle sizes were examined in this study. The volume of 6 μm particle size was 25, 45, and 60 (measured in wt% units), and for 90 nm particles, it was 25 and 45 wt%, as the volumes higher than 45 wt% were not miscible in nanopowder form. The sensing properties of their MoS_2_ electrodes were evaluated in a 0.1 M phosphate buffer solution (pH 7.0) with the presence of dopamine. The DPV curve was used to determine the electrochemical oxidation of dopamine and the linear dependance of oxidation peak current vs. dopamine concentration in the range up to 300 μM. The authors reported the performance of 6 μm particles of MoS_2_ and a volume of 45 wt% (Mo6-45) gave the best performance in response to dopamine presence with LOD value of 246 nM and sensitivity of 5.00 × 10^−8^ A μM. The other modified MoS_2_ electrodes’ performance is summarized in Table 1.

**Figure 5:**
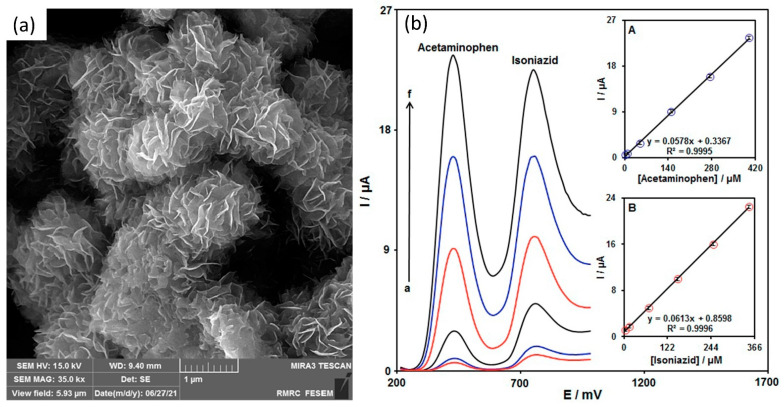
(**a**) FESEM image of MoS_2_ nanosheets synthesized in Tajik et al.’s laboratory. (**b**) Differential pulse voltammetry of simultaneous detection of INZ and AC on MoS_2_-NSs/SPE. Inset (**A**): The anodic peak current plot vs. AC concentration. Inset (**B**): The anodic peak current plot vs. INZ concentration. Reprinted with permission from [114]. Copyright 2022 MDPI. Authors licensed under a Creative Commons Attribution (CC BY) license.

Owing to its large surface area, 2D MoS_2_ material is frequently used on the WE of electrochemical sensor for direct detection. Asefifeyzabady et al. [103] carried out a study of MoS_2_-based electrochemical sensor for detection of trinucleotides CGG repeats, which are crucial for combating a variety genetic disorders. It is a three-electrode system, where the counter and pseudo electrodes were inkjet-printed with silver nanoparticles (AgNPs), and the WE was drop-casted with their laboratory prepared MoS_2_ solution on top of the AgNPs electrode. The detection of the CGG on the MoS_2_ WE surface relied on the current response in the presence of [Fe(CN_6_)]^−3/−4^ using DPV analysis method. In their paper, Asefifeyzabady et al. discovered two important aspects of the DNA detection on the MoS_2_ sensor surface. First, this study identified that the direct adsorption of DNA on the MoS_2_ enhanced the electrocatalytic current with the number of CGG repeats length. This finding is pivotal and can be used to distinguish between normal and abnormal repeats length. Second, they found that the DNA duplex conformation would remain on the MoS_2_ surface after hybridization and give rise the electrocatalytic current, resulting in an increase in the DPV oxidation. In contrast to previously published studies, Chu et al. [104] and Loo et al. [105] demonstrated a decrease in the oxidation peak current value of the DPV signal because the double-stranded DNA (dsDNA) formed by hybridization of probe DNA (pDNA) and complementary DNA (cDNA) had been released from the MoS_2_ electrode surface. The proposed MoS_2_/SPE sensor exhibited successful detection of double-stranded CGG-8 (dsCGG-8) repeats using DPV analysis in the concentration range of 1 aM–100 nM and showed a good linear calibration curve (R^2^ = 0.992) with 3.0 aM LOD. Zribi et al. [106] described a voltammetry technique for measurement of tyrosine by employing SPCE and screen-printed gold electrodes (SPAuE) modified with MoS_2_ nanomaterial. In it, 0.45 μg/mL of MoS_2_ dispersion was directly drop-cast on top of both SPE working electrodes as a modifier. The CV analyses on both SPCE and SPAuE showed that the tyrosine oxidation current peak intensity was enhanced proportionally to its concentration. On both modified SPEs with MoS_2_, the current signal was observed to enhance owing to the intrinsic electrocatalytic activity of 2D-MoS_2_ [107]. The sensitivity of the SPCE and SPAuE sensors was 1400 μA·mM^−1^·cm^−2^ and 1580 μA·mM^−1^·cm^−2^, respectively, in the linear range of 0–100 μM. The LOD of the SPCE sensor was determined to be 31.23 μM, while LOD of the SPAuE sensor was 0.5 μM. Furthermore, the authors also carried out a chronoamperometric analysis to determine tyrosine with MoS_2_-modified SPCE under a constant voltage (0.6 V vs. Ag/AgCl). They reported that the oxidation current rapidly risen-up with tyrosine concentrations up to 617 μM. The current-concentration curve exhibited dynamic linearity in the very low concentration range (0–50 μM), provided sensitivity of 148 μA·mM^−1^·cm^−2^ with LOD of 1.4 μM. However, the present study has suffered from inadequate data. The authors only examined the detection of tyrosine on MoS_2_-SPCE sensor, there are no amperometric data reported for tyrosine detection on MoS_2_-SPAuE sensor device. Further research could usefully explore the performance of MoS2-SPAuE sensor in tyrosine detection using chronoamperometry analysis. The group reported further development of MoS2-SPCE sensor for simultaneous detection of dopamine (DA) and tyrosine (Tyr) in the presence of uric acid [108]. Similar to previous work, Zribi et al. modified SPCE with MoS_2_ via direct drop-casting method on the WE. The detection of DA and Tyr was done in a buffer using cyclic voltammetry (CV) and linear sweep voltammetry (LSV) analysis. Data from CV analysis shows separation oxidation peak current of DA and Tyr at 0.16 V and 0.57 V, respectively. They also investigated current-concentration dependence of the SPCE on both the DA and Tyr target samples using LSV analysis technique. Both detections demonstrated linear fit in the range of 0–100 μM for DA and 0–500 μM for Tyr. The limit of detection for DA and Tyr was 0.085 μM and 0.5 μM, respectively. Further, the investigation of simultaneous detection of DA and Tyr has been carried out in the presence of uric acid (UA) using LSV technique. Their findings show three distinct peaks at different voltages. Additionally, they also reported LSV data with variations of UA, DA, and Tyr concentrations to examine the dependency of DA and Tyr detection in UA presence. Their result revealed that the detection of DA and Tyr was almost independent with or without the presence of UA. Tajik et al. synthesized MoS_2_ nanosheets (MoS_2_-NSs) using a single-pot hydrothermal protocol and dispersed them on SPE to fabricate a MoS_2_-NSs sensor to detect isoniazid (INZ) and acetaminophen (AC). INZ is a commercially available medication that is routinely given as a treatment for tuberculosis (TB) patients. Long-term exposure to INZ may induce hepatotoxicity in people suffering from inflammation and may even cause fatality [109,110]. An overdose of AC (4 g/day) can lead to hepatotoxicity, nephrotoxicity, gastrointestinal problems, and tissue failure [111]. The main motivation of Tajik’s work was based on the growing concern of liver damage because of INZ co-administered with AC. The prepared MoS_2_/SPE possessed excellent electrochemical properties with high current response and large active surface area of 0.16 cm^2^ which was 5.1 times larger than the bare SPE. Figure 5a shows the FESEM image of the synthesized MoS_2_ nanosheets. Accompanied by high catalytic activities and large active sites, the MoS_2_/SPE sensor demonstrated LOD of 10 nM and exhibited linear elevation with a wide range of INZ concentration (0.035–390 μM). In this work, Tajik also demonstrated simultaneous detection of INZ and in the presence of AC. Two separated and well-defined oxidation peaks were observed using DPV method, as shown in Figure 5b, suggesting that the MoS_2_/SPE sensor potentially allowed for simultaneous detection. It can be seen that the sensor has good stability, as the peak current of INZ remained stable even after 4 weeks. Furthermore, the sensor showed a good mean relative standard deviation (RSD) value, capable of real sample analysis. In a separate study, Neethipathi et al. proposed a MoS_2_/SPE for heavy metal ions copper (Cu^2+^) detection [112]. The electrochemical electrode was printed using carbon paste by Screen Stencil Printer C920 (AUREL Automation) on flexible polyvinyl chloride (PVC) substrate. Sensing area modification was done by drop-casting solution of MoS_2_ in dimethylformamide (DMF) on the working electrode. Using DPV analysis, the sensor successfully recognized the Cu^2+^ in the linear concentration range of 5 μM to 1000 μM with LOD of 0.3125 μM. Additionally, the sensor showed excellent stability and repeatability by exhibiting a very small current deviation (less than 0.5%) during the course of 40 continuous cycles of CV analysis in PBS solution containing 100 μM of Cu^2+^. Due to its robustness, this sensor provides an optimistic future route for the electrochemical sensor development for heavy metal ions in harsh industrial waste. Compared to the recently published article on single- and multi-walled carbon nanotubes (SWCNTs and MWCNTs) modified SPE for the detection of quinone-based industrial pollutants, the proposed sensor by Neethipathi et al. was inferior in terms of sensitivity. The SWCNTs and MWCNTs modified SPE had lower LOD values of 0.04 μM and 0.07 μM, respectively [113]. However, it must be noted that the comparison is established only on the sensitivity of the sensing materials for industrial pollutant detection in general. Further exploration of MoS_2_ potential for the detection of quinone-based industrial pollutants is essential for a more fair comparison.

Efficient MoS_2_ nanocomposites combined with SPE designed for low-cost manufacture could lead to a potentially commercially viable, reproducible, and sensitive sensor device. This has been demonstrated in recent years by Demir et al. [115], who used SPE based on MoS_2_-TiO_2_/rGO nanocomposites to determine AC in paracetamol using DPV for signal interpretation. Following the common technique that has been used by many researchers, Demir et al. prepared the SPE for AC detection by drop-casting dispersion of MoS_2_-TiO_2_/rGO on the working electrode. They reported that the DPV signal (current peak) of MoS_2_-TiO_2_/rGO/SPE was linearly proportional to the concentration of AC in the range of 0.1 μM to 125 μM. The LOD of the sensor was 0.046 μM. Moreover, their sensor also surpassed the sensitivity of most other sensors that have been reported in the literature for detection of AC with sensitivity value of 0.4425 µA µM^−1^. The excellent sensing performance demonstrated by this sensor was attributed to MoS_2_-TiO_2_/rGO nanostructure. The MoS_2_ sandwich structures have provided unsaturated edge sites for surface functionalization [116], and the regulation band structures, consequently boosted the sensor performance [117], while TiO_2_ has been utilized in the composition to enhance the electrocatalytic effect on the electrode surface [118], coupled with rGO, which served for efficient electron transfer and high surface area for sensing purposes [119]. Beyond that, there are another few likely reasons that contributed to such excellent sensing performance: (1) interfacial properties of MoS_2_ nanoparticles with rGO sheets in the structure of composite create a synergetic effect, (2) high electrical conductivity of rGO nanosheets, and (3) enhancement in the surface area leading to an increment in the electrocatalytic activity of MoS_2_-TiO_2_/rGO owing to inhibition of nanomaterial aggregation. Their sensor exhibited excellent selectivity and sensitivity as well as recovery for direct diagnosis of AC in drug and real human urine samples. Gui et al. developed chemically exfoliated MoS2(ce-MoS2)/silver nanorods (AgNRs)-modified SPE immunosensor for prostate-specific antigen (PSA) detection [120]. In this study, negatively charged ce-MoS_2_ and positively charged AgNRs dispersion were integrated onto the SPE working electrode to form a nanostructured surface, in which the anti-PSA antibodies were directly immobilized. It is worth mentioning that the AgNRs were anchored onto the MoS_2_ nanosheets’ surface via cetyltrimethylammonium bromide (CTAB), which altered the AgNRs’ electrical properties and, accordingly, expedited amalgamation of AgNRs on MoS_2_ nanosheets via electrostatic interaction. The electrochemical impedance spectroscopy (EIS) data analysis exhibited a wide detection range of 0.1 to 1000 ng mL^−1^ with high reproducibility and good stability for 21 days at 4 °C. The detection limit of this MoS_2_/AgNRs sensor was 0.051 ng/mL. Another interesting SPE biosensor using MoS_2_-based nanocomposite was reported by Sadeghi et al. [121]. This biosensor was designed with PANI/MoS_2_ nanocomposites as sensing materials for molecular identification of Guanine (G), Adenine (A), Thymine (T), and Cytosine (C) nucleobases (DNA nucleobases). The research group performed a simultaneous detection of G, A, T, and C nucleobases in quaternary solutions and reported the DPV oxidation peak currents of all the DNA nucleobases were approximately linear. The detection of G and A were linear in the range of 0.1–10 μM, while the detection of T and C also demonstrated good linear results, but in a different range (1.2–100 μM). However, the linear regression of calibration curves showed inconsistency when the G, A, T, and C nucleobases were quantified individually. According to the authors, these findings may reflect the change in control factor of the sensing electrode surface. As for G and A nucleobases, the surface reaction at low concentration was controlled by diffusion processes, while at high concentration, the surface reaction was governed by the absorption process. In contrast to C nucleobase, an inverse finding was observed. The research, however, is limited by the lack of information and clarity on the individual determination of T nucleobase. The LOD of G and A was 0.015 mol L^−1^ when quantified individually as well as in a quaternary solution, while the LOD of T and C was reported to be 0.14 mol L^−1^ when quantified in both conditions. This PANI/MoS_2_-nanocomposite-based biosensor revealed noteworthy electrocatalytic activity for G and A nucleobase oxidation as it demonstrated the highest peak oxidation current compared to bare CPE, MoS_2_/CPE and PANI/CPE. This could be attributed to the strong π-π* interaction and electrostatic adsorption, leading to an efficient electron change between the free bases and the electrode surface. The biosensor confirmed its stability and reproducibility with RSD for quantification of A and G of 4.06% and 3.76%, respectively. In another study, graphene was mixed with MoS_2_ and dispersed together to create a nanocomposite for determination of trans-resveratrol (TRA) in red wine using SPE sensor [122]. In this approach, MoS_2_ layer was synthesized on the surface of graphene layers to form sheet-on-sheet graphene-MoS_2_ (Gr-MoS_2_) nanocomposite, which was later deposited on top of the SPE working electrode. Here, the Gr-MoS_2_ acts as a matrix for direct sensing of TRA from a wine sample. It should be noted, however, that the TRA sample required pre-treatment, such as extraction from wine, before being electrochemically quantified using this sensor. Using square wave voltammetry (SWV) technique, the sensor successfully recognized the TRA concentration from 1.0 to 200 μmol L^−1^ with 0.45 μmol L^−1^ LOD.

**Figure 6:**
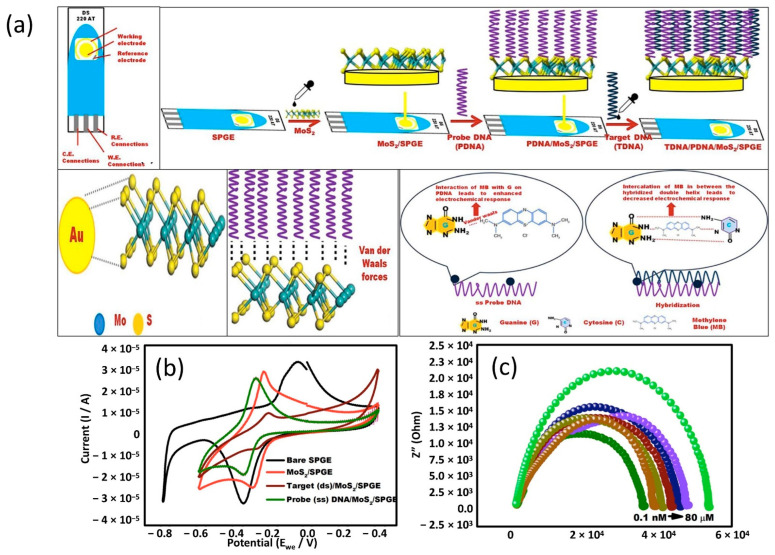
(**a**) From left to right, fabrication steps of MoS_2_/SPGE sensor for chikungunya virus. Stepwise shows fabrication of SPGE with MoS2 nanosheets, immobilization of probe DNA and target DNA. The next illustration shows strong affinity interaction of Au and S (in MoS_2_), Van der Waals interaction between probe DNA and the MoS_2_ surface, principle for the detection of target DNA via redox hybridization indicator, i.e., methylene blue. (**b**) Comparison of cyclic voltammetry graph of bare SPE, MoS_2_/SPE, Target/MoS_2_/SPE, and Probe/MoS_2_/SPE. (**c**) Nyquist Plot verifying hybridization of the different concentration of the complementary target DNA at PDNA/MoS_2_NSs/SPGE. Reprinted with permission from [133]. Copyright 2018 Springer Nature. Authors licensed under a Creative Commons Attribution (CC BY) license.

Turning now to the experimental evidence on decorated MoS_2_ as a sensing material. Generally, MoS_2_ exists as two kinds of crystalline phases: 2H phase (2H-MoS_2_) and 1T phase (1T-MoS_2_). The 1T-MoS_2_ has been reported to exhibit higher conductivity and superior charge transfer ability, attributed to its metallic property [123]. Moreover, it also possesses higher catalytic activity because both the edges and basal plane are catalytically active [124]. For this reason, modification of electrochemical sensor with 1T-MoS_2_ has demonstrated excellent sensing performance, surpassing that of electrochemical sensor modified with 2H-MoS_2_ [125]. Despite its advantages for electrochemical applications, 1T-MoS_2_ is a metastable material, and its mechanisms for stable performance are yet to be clarified [126]. In contrast to 1T-MoS_2_, 2H-MoS_2_ has more stability with semiconductive property [127]. However, it suffers from low density of active sites and poor electrical transport property. Experimental evidence has established that the catalytically active sites of 2H-MoS_2_ monolayer are generally located only at the edges and corners, whereas its basal atoms are catalytically inert [128], consequently limiting the catalytic performance of the 2H-MoS_2_. Er et al. carried out a study on 1T-MoS_2_ decorated with shape-dependent gold nanostructures for determination of doxorubicin (DOX) [129]. In this work, a SPE surface was modified with single-crystal gold nanospheres (AuNSPs) and nanorods (AuNRDs). These golds were decorated separately on 1T-MoS_2_ layer on top of the SPE WE and their electrocatalytic activity towards DOX was analyzed. Taking an evidence-based approach, Er et al. showed that the 1T-MoS_2_/AuNRDs exhibited better electrocatalytic efficiency as a sensing material compared to 1T-MoS_2_/AuNSPs. Their CV analysis revealed that the SPE with 1T-MoS_2_/AuNRDs had the narrowest peak-to-peak separation (ΔE_p_) and larger anodic and cathodic for Fe (CN_6_)^−3/−4^ compared to bare SPE and 1T-MoS_2_/AuNSPs/SPE, implying that the 1T-MoS_2_/AuNRDs has the fastest electron transfer capability between probe molecules and the SPE surface. It is noteworthy that the redox peaks that have been observed corresponded to the oxidation of hydroquinone and the reduction of quinone groups that occupied the center of DOX structures [130]. Furthermore, the 1T-MoS_2_/AuNRDs/SPE sensor exhibited high sensitivity of 7.13 μA μM^−1^ cm^−2^ towards the DOX assay and excellent LOD (2.5 nM) with a linear range between 0.01 to 9.5 μM. The sensor has also shown remarkable selectivity in real samples analysis. This sensor was successfully used for direct detection of DOX in human blood serum without pre-treatment. In another study, 2H-MoS_2_ nanosheets (MoS_2_ NSs) decorated with controlled density of mono dispersed small gold particles (AuNPs@MoS_2_) for detection of miRNA was reported [131]. Here, the sensing strategy that was used in this approach was upon hybridization of capture probe ssDNA (CP) with target miRNA sequence (TR), alongside with complementary ssDNA sequence (AP) that served as signal amplification. These elements were mainly immobilized into the AuNPs@MoS_2_ screen-printed gold electrode (SPGE) transducer by absorption. The quantification of miRNA was done by chronocoulometry (CC) technique based on the number of cationic redox molecules [Ru(NH_3_)^6^]^3+^ that were electrostatically associated with the anionic DNA backbones. In a study conducted by Steel et al. [132], it was shown that one [Ru(NH_3_)^6^]^3+^ cationic redox marker was electrostatically trapped for every three nucleotide phosphate groups. This study also assesses the significant differences in thickness and size of MoS_2_ platelets. They discovered that the AuNP@MoS_2_/SPGE sensor with the thinnest and smallest MoS_2_ nanosheets has superior performance compared to larger and thicker MoS_2_ nanosheets. The LOD of AuNP@MoS_2_/SPGE sensor with the thinnest and smallest MoS_2_ nanosheets was down to 100 aM, which is 2 orders of magnitude lower than bare gold (Au) electrode. In addition to that, the sensor also demonstrated a 25% enhancement in DNA-miRNA hybridization efficiency. Singhal et al. and co-workers carried out a series of experiments to investigate potential of MoS_2_ on screen-printed gold electrode sensor (MoS_2_/SPGE) to detect chikungunya virus [133]. This prototype used hybridization of DNA on the MoS_2_/SPGE as a determinant for sensor detection. The fabrication of the DNA-based sensor for chikungunya virus detection on MoS_2_/SPGE was demonstrated as in Figure 6a. Probe DNA was simply immobilized on the SPGE sensing areas that were coated with MoS_2_, followed by target DNA and methylene blue (MB). The addition of MB in this experiment step allowed for electrochemical response enhancement. The biosensor response was recorded using voltammetry technique, where they observed that the potential was shifted to the negative side with immobilization of the probe DNA and reverted to the positive side upon hybridization with the target DNA, as shown in Figure 6b. This finding is likely to be related to the negatively charged nature of DNA and electron transfer during the hybridization process [134]. This phenomenon can be explained as follows. The MoS_2_ surface and MB undergo a redox reaction during cyclic voltammetry to produce the characteristic voltammogram at a specific potential. Immobilization of probe DNA on MoS_2_ surface altered the electron transfer, resulting in decreased potential and making the MB easier to reduce (potential shifted to the negative side). When the target DNA was added onto the MoS_2_ surface, hybridization occurred, causing restructuring molecules, and called for more electrons to reduce the MB. However, fewer electrons were available for reducing MB, resulting in an increased reduction potential (potential reverting to the positive side). The biosensor’s performance was validated by quantifying the target oligonucleotides in a serum sample and was shown to be a successful detection via EIS technique (Figure 6c). The LOD of this biosensor was 3.4 nM with a linear range of 0.1 nM to 100 μM.

**Figure 7:**
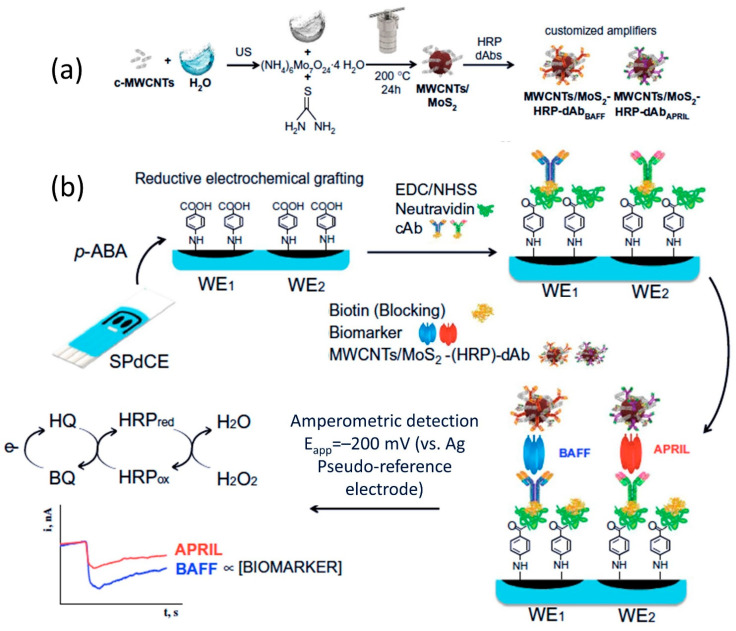
(**a**) A schematic of the fabrication of MWCNTs/MoS2(-HRP)-dAb nanocarriers and (**b**) their application on screen-printed dual carbon electrodes (SPdECs) for the determination of BAFF and APRIL biomarkers. Reprinted with permission from [135]. Copyright 2022 Springer Nature. Authors licensed under a Creative Commons Attribution (CC BY) license.

As a different approach, MoS_2_ also has been utilized as biomarker labels for signal amplification, to improve sensitivity of an immunosensor. Arévalo et al. [135] have integrated this concept into their amperometric multiplexed immunosensor for the determination of B cell activation factor (BAFF) and proliferation-induced signal (APRIL) immunity-related cytokines. In this work, screen-printed dual carbon electrodes (SPdECs) are modified with the biotinylated capture antibodies cAB_BAFF_ and cAB_APRIL_ on their respective working electrodes. This proposed sensor employs sandwich-type modes of operation, where a layer of antibody binds with analyte-antigen, which then binds with second labeled-antibody. A major advantage of this mode of operation is that it allows for measuring large antigens that are capable of binding two different antibodies. Both biomarkers BAFF and APRIL were detectable in buffer at concentrations ranging from 0.24 to 120 ng mL^−1^ (r^2^ = 0.999) and 0.19 to 25 ng mL^−1^ (r^2^ = 0.997), respectively. The dual-immunosensor provided theoretical limit of detection of 0.08 for BAFF and 0.06 ng mL^−1^ for APRIL. The sensor device has excellent selectivity and recognized both cytokines in cancer cell lysates and serum samples (diluted 10 times) from patients diagnosed with autoimmune diseases and cancer. In this investigation, Arévalo et al. employed MoS_2_ as biomarker carrier tags instead of WE (sensing area) modifier. The role of MoS_2_ in this work was specifically for carrier labels for signal amplification to improve the immunosensors’ sensitivity. For a proper understanding of this sensor detection principle, a schematic of the fabrication sensor protocol is shown in Figure 7. Other works of MoS_2_ on printable sensor are summarized in Table 2.

### 4.2. Molybdenum Diselenide (MoSe_2_)

Similar to any other TMDCs material, molybdenum diselenide (MoSe_2_) exists in a sandwich structure, which comprises of one Mo covalently bonded with two chalcogen atoms of selenide (Se), and each monolayer is held by a weak force. Compared to its counterpart (MoS_2_), MoSe_2_ has superior electrocatalytic reaction and specific conductance on account of twenty orders greater metallic conductivity, more functional unsaturated sites of selenides, attractive energy density, a faster electrochemical reaction rate, higher metallic binding with transition metals, and a larger atomic size [147,148,149]. With these fascinating properties, MoSe_2_ has been widely used in various electrochemical applications [150,151,152].

A recent study reported the application of MoSe_2_ on SPE as a disposable and portable sensor for detection of hydrogen peroxide (H_2_O_2_) in ultra-wide pH [153]. Other than the extraordinary properties listed above, MoSe_2_ also has a small bandgap (~1.2 eV) and large interlayer spacing, which particularly favors the reduction of H_2_O_2_ [154,155]. However, in spite of the great electrochemical properties, the MoSe_2_ basal plane generally has poor conductivity and lack of active sites, which thereby hinders binding with -OH intermediate. To overcome this challenge, defect engineering and heteroatomic doping strategies were used to introduce strain and vacancies on the MoSe_2_ basal plane. The MoSe_2_ was synthesized by hydrothermal method, and further introduced to argon (Ar) and nitrogen (N_2_) plasma treatments to create new active sites to enhance its catalytic performance. The defect MoSe_2_ evidently has stronger -OH binding and on its surface when the SPE sensor has accomplished higher sensitivity and selectivity. For H_2_O_2_ sensing, the SPE sensor was operated amperometrically at −0.30 V vs. saturated calomel electrode (SCE). The finding shows that the current response curve was linearly increased with H_2_O_2_ concentration in the range of 0.02–0.2 mmol/L (at low concentration) and 0.2–20 mmol/L (at high concentration). This electrical signal response was generated from the reduction of H_2_O_2_ to H_2_O. The sensitivity of the sensor was calculated to be 16.22 µA/(mM·cm^2^) and 15.68 µA/(mM·cm^2^) at low and high concentrations, respectively. This sensor achieved detection limit of 462.4 nmol/L. In separate work, Ramaraj et al. performed a series of experiments to show the doping effect of MoSe_2_ in sensing applications, and further reported an iron (Fe)-doped MoSe_2_/SPE sensor for mesalamine (MES) determination, an anti-inflammatory agent in paramedical sample [156]. The MoSe_2_ was doped with Fe using three different synthesis methods, which were hydrothermal, microwave-assisted synthesis, and chemical synthesis. Each of the Fe-doped MoSe_2_ products were drop-casted onto separate SPEs to assess their electrochemical performance. SPE that is drop-casted with Fe-doped MoSe_2_ via hydrothermal, microwave-assisted, and chemical synthesis is denoted as H-FeMoSe_2_/SPE, M-FeMoSe_2_/SPE, and C-FeMoSe_2_/SPE, respectively. From their findings, H-FeMoSe_2_/SPE has the highest electrocatalytic, superior sensing performance, and lowest detection limit compared to M-FeMoSe_2_/SPE and C-FeMoSe_2_/SPE. The relevance of these enhanced sensor performances was clearly supported by their EIS and CV data, where the H-FeMoSe_2_ has the lowest R_CT_ value, suggesting faster electron transfer and the highest current response, as well as the narrowest peak-to-peak separation in CV analysis, attributed to improved charge transfer conductivity. The determination of MES was analyzed by CV technique. The sensing mechanism relied on the conversion of MES to quinone-imine. As they concluded, H-FeMoSe_2_/SPE has the sharpest and highest oxidation peak current response in comparison to the other two FeMoSe_2_/SPE (M-FeMoSe_2_/SPE and C-FeMoSe_2_/SPE). This result may reflect differences in layer structure, thickness, and quality of the Fe-doped MoSe_2_. Moreover, the oxidation peak current was linearly increased with MES concentration in the ranging from 0.09 to 0.654 mM in N_2_ purged buffer (pH 7). The selectivity of the sensor was also excellent as it yielded less than 5% current change with the presence of interferences. This H-FeMoSe_2_/SPE was tested for its ability to determine the presence of MES in Pentasa tablet. The analysis is done using DPV method, and they found out that the sensor was capable of quantifying MES in Pentasa solution, with 101.1–104% recovery and RSDs ranging from 2.1 to 2.7%.

### 4.3. Tungsten Disulfide (WS_2_)

One of the most important constituents in any sensor device is a large surface area with very a good sensing matrix, and in recent years, the 2D material tungsten disulfide (WS_2_) has attracted considerable attention for this kind of application. WS_2_ is a promising material candidate for future electronic and optoelectronic sensor applications owing to its unique combination of outstanding properties such as its 2D ultrathin atomic layer, high surface area, and good biocompatibility [157,158]. Furthermore, WS_2_ also accommodates for self-assembly of thiolated compounds on its surface, which makes it more attractive compared to graphene material in terms of effortless surface modification [159]. WS_2_ is one of the materials that belongs to the 2D TMDC group, in which the tungsten (W) layer is sandwiched between two sulfur layers (S) and stacked together by weak Van der Waals forces [160].

**Figure 8:**
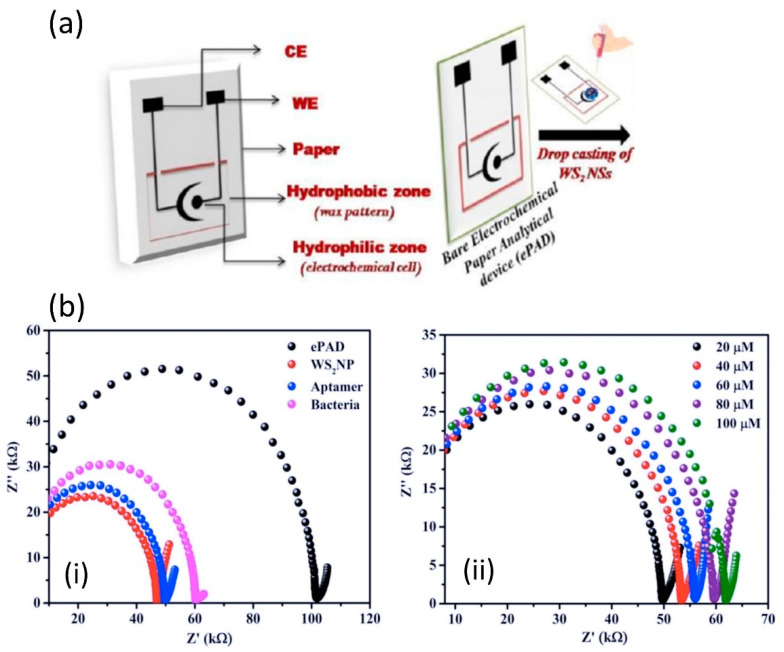
(**a**) Fabrication of screen-printed sensor on paper-based substrate is also well-known as paper-based analytical device (ePAD) and (**b**-**i**) Nyquist plot of bare ePAD, ePAD/WS_2_NS, after immobilization with aptamer and after immobilization with bacteria, (**b**-**ii**) Nyquist plot of detection bacteria at different concentration ranging from 20 μM to 100 μM. Reprinted with permission from [161]. Copyright 2021 MDPI. Authors licensed under a Creative Commons Attribution (CC BY) license.

**Figure 9:**
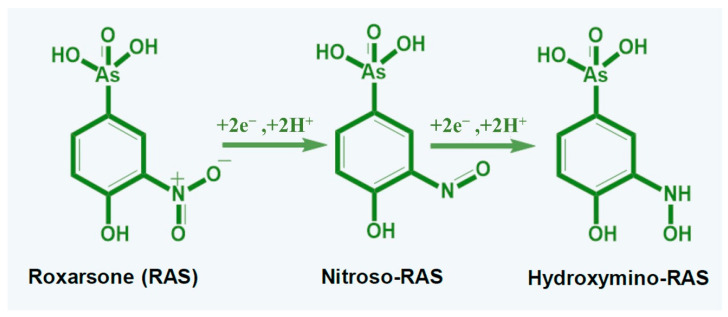
An illustration of electrocatalytic reaction mechanism of toxic roxarsone on the WS_2_-based SPE electrode sensor. Reprinted with permission from [167]. Copyright 2019 Elsevier.

Mishra et al. developed an electrochemical paper-based analytical device (ePAD) based on WS_2_/aptamer hybrid, which provided a very selective approach for *Listeria monocytogenes* determination [161]. The ePAD was constructed on paper by screen-printed conductive carbon ink using silk mesh as a sensor pattern stencil. Different from any other printed sensor that has been reviewed in this article, Mishra’s work presented an ePAD sensor with only two electrodes, WE and CE. The sensing matrix on this sensor prototype was quite straight forward; WS_2_ was drop-casted onto the WE and left to dry before aptamer immobilization. The incorporation of aptamer as a biological recognition element provides specificity for the impedimetric assay. Figure 8a exhibits the development of two electrode sensor on a paper substrate (ePAD), and Figure 8b demonstrates an ePAD sensor competitive assay for *Listeria monocytogenes* that was recorded with EIS technique. The EIS assessment data of this sensor revealed a correlation of *Listeria monocytogenes* concentration with the sensor’s surface sensitivity, which provides a linear range of 10^1^–10^8^ CFU/mL and a limit of detection of 10 CFU/mL. The use of WS_2_ in combination with other functional materials as a sensing matrix offers the possibility of a new generation of highly specific, sensitive, selective, and reliable sensors for various detection. One practical work that combined not only one, but two functional materials with WS_2_ as a sensing element was recently discussed by Pelle et al. [162]. The research group capitalized on the superior sensing layer of 2D WS_2_ flakes decorated with catechin-gold capped nanoparticles (AuNPs-CT) and a carbon black-based screen-printed sensor for simultaneous determination of structural analogs of hydroxycinnamic acid (hCN); caffeic (CP), sinapic (SP), and *p*-coumaric acids (CM). The functional material, such as catechin-capped gold nanoparticles (AuNP-CT) was exploited to provide higher sensitivity and specificity, while the carbon black served as high conductivity and high electroactivity material. With regard to WS_2_ flakes, they play a vital role in enhancing sensor performance by increasing the sensing surface area and electron transfer. Moreover, it is well-known for antifouling properties towards flavonoid’s oxidation [163]. The CV and DPV were used for the analysis of the interaction of the hCNs targets with the CB-WS_2_/AuNPs-CT sensor surface. Their DPV results showed individual detection of CF, SP, and CM, while simultaneous detection of CF, SP, and CM generated three oxidation peaks at three different potentials (110 mV, 330 mV, and 490 mV). The data were analyzed by following polyphenol (PC) antioxidant capacity series to discriminate the three oxidation peaks. For example, (i) CF has a higher antioxidant capacity due to its ortho di-phenols structure and was oxidized at the lowest potential (110 mV). (ii) SP has an Intermediate antioxidant capacity with monophenols with a 4-hydroxy-3,5-dimethoxy structure, was oxidized at the intermediate potential (330 mV) and (iii) CM has the lowest antioxidant capacity among the three phenols with monophenols structure and was oxidized at the highest potential (490 mV). The results of this study were in line with earlier literature [164,165] that found that the antioxidant capacity series of polyphenols follows the trend of o-diphenols > methoxy-substitute monophenols > monophenols. The sensor showed a good limit of detection of 0.1 μmol L^−1^, 0.4 μmol L^−1^, and 0.4 μmol L^−1^ for detection of CF, SP, and CM, respectively. It is worth mentioning here that this sensor has long-term stability. In a controlled condition, for the first 2 months, the response of the sensor decreased to 95%, however, it remained stable for the next 4 months (the sensor response was about 94%). It also successfully demonstrated simultaneous determination of CF, SP, and CM in a food sample with good precision (RSD ≤ 4%, *n* = 3). A different study reported the integration of WS_2_ nanosheets with SPE for coccidiostat drug (roxarsone) determination that is widely used in poultry farming [166]. The roxarsone electro-reduction on the WS_2_/SPE electrode was concluded as an irreversible process, where the NO_2_ was reduced to NH-OH (as shown in Figure 9), accompanied by a reduction peak potential at −0.64 V. The high current response has also been observed to be higher than electro-reduction roxarsone on bare SPE. Good catalytic performance and porous properties of WS_2_ nanosheets could be the contributing key factors for the high current response. In a concentration-dependent experiment, the reduction peak of roxarsone increased successfully with the roxarsone concentration and showed an extremely good linear response in the range of 0.05 to 489.3 μM. LOD of this sensor was 0.03 μM. The sensor was also implemented for the determination of roxarsone in real meat samples, demonstrating accuracy of 96.29% and 112.5% in chicken extract and pork extract, respectively.

## 5. MXenes

**Figure 10:**
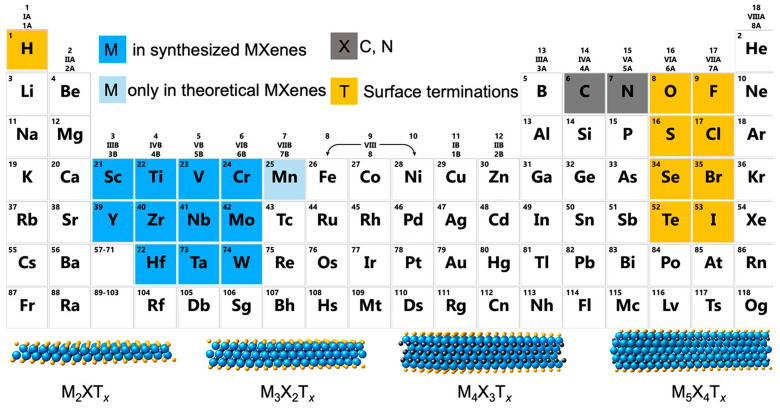
Periodic table presenting elements that are used in MXenes’ composition. At the bottom are illustrations of four typical MXene structures. Reprinted (adapted) with permission from [172]. Copyright 2021 American Chemical Society.

MXenes are a new class of two-dimensional material that has been proven to be valuable in various electronic fields, including biosensors, since its discovery in 2011 by Naguib et al. [168]. MXenes material is based on transition metal carbides and nitrides with a chemical formula of M_n+1_X_n_T_x_, where M represents a transition metal, X is an element of carbon or nitrogen, and T_x_ is denoted as termination on the surface of the outmost transition metal layers. The MXenes elements and four typical structures of MXenes materials are shown in Figure 10. Over the years, various MXenes have been demonstrated in literatures, however, only Ti_3_C_2_ and Ti_2_C are widely used in electronic applications. The discovery of MXenes materials about ten years ago opened another door of opportunity for sensing technology. MXenes offers high electrical conductivity (15 100 S cm^−1^) owing to the metal component in its structure [169]. Essentially, MXenes have a negatively charged surface that mostly comprises hydroxyl (-OH), fluorine (-F), and oxygen (-O) groups [170]. These functional groups on MXene’s surface are attributed to chemicals used during MXene’s synthesizing process. Under those circumstances, this negatively charged surface can provide favorable reaction sites for positively charged molecules, for instance, heavy metal ions such as aluminum (Al^3+^), silver (Ag^+^), and copper (Cu^+^). Besides that, MXene’s work functions are tunable by controlling electron density. On that account, with appropriate terminal groups, MXene’s surface can be exploited for sensing certain analytes [171].

The unstable nature of enzymes has restricted the use of enzymes in any sensor application. Immobilization of enzymes on substrate surfaces typically results in decreased enzyme activity and impaired efficiency [173]. Significant driving factors for the reduction in enzyme activity include binding procedure, diminished availability of enzyme molecules within substrate pores, or slowly diffusing substrate molecules. These factors are together known as mass transfer effect. Recently, Wu et al. [174] capitalized on MXenes material to develop a stable enzyme-based electrochemical sensor for detection of the D-2-hydroxyglutaric acid (D2HG) in serum and urine. *Ralstonia solanacearum* (*Rs*D2HGDH) enzyme, used in this work as an ultrasensitive sensing element, was co-immobilized with methylene blue (MB) on Ti_2_C_3_ MXenes sheet and further drop-cast onto the AuSPE with the help of chitosan. The research group reported that the stability of the *Rs*D2HGDH enzyme was maintained even after 60 h immobilized on the MXenes surface. It is plausible that the *Rs*D2HGDH benefited from the abundant functional groups of the MXenes Ih provided a favorable environment Ior its immobilization. Research findings by Liu et al. also point towards functional groups that interact with the enzyme through weak interactions could stabilize the native structure while retaining the enzyme activity [175]. The detection of the sensor was based on the electron transfer between *Rs*D2HGDH and the gold electrode. In the presence of D2HG, *Rs*D2HGDH will be catalyzed and generate electrons. These electrons were shuttled through the MB mediator to the sensor electrode. The prepared *Rs*D2HgDH-MXenes-AuSPE biosensor showed detection of D2HG in the range of 0.5 to 120 µM, with LOD of 0.1 μM. The sensitivity of the biosensor was 22.26 µA mM^−1^ cm^−2^. The authors claimed that their AuSPE sensor gave better performance compared to the commercial D2HG assays kit, with LOD of 10 μM and essentially needs controlled temperature when operating. Nevertheless, this sensor system suffers a drawback from the use of the mediator. They reported that the oxygens were competing with the mediators in some experiments, while the stability and biological toxicity of the mediators need further investigation. Therefore, to overcome the problem with mediator, further studies on new D2HGDH that can bypass the mediated electron transfer is essential.

A valuable advantage of SPE sensors is that their sensing element can be printed directly on a substrate, consequently, remove complexity of the sensor device’s fabrication. Moreover, direct printable sensing materials can minimize sensor variation with controlled film thickness and hold great potential for mass production. Saleh et al. and his research group developed water-based MXenes ink and inkjet-printed MXenes film on freestanding poly(3,4-ethylenedioxythiophene):polystyrene sulfonate (PEDOT:PSS), that is suitable for wearable printed sensors [176]. Of note, the MXenes material has shown outstanding hydrophilicity and biocompatibility with biological tissue, making it an ideal candidate for a skin-contact sensor. Owing to PEDOT’s conductive properties, the entire MXenes sensor fabrication required only a single printing step. The metallic connection underneath the MXENEs electrode was unnecessary. This is because PEDOT allowed for a conducting, metal-like contact point to external acquisition system. The prepared MXenes electrode was tested for recording electrocardiography (ECG) signals from skin. Generally, commercial Ag/AgCl gel-type electrodes have been used to measure such signals. Therefore, it is reasonable to compare the performance of the MXenes electrode with the commercial Ag/AgCl electrode for ECG signal recordings. Their findings showed that the impedance between MXenes and the human skin was lower than the impedance of the Ag/AgCl electrode, suggesting conformability of the MXenes film electrode on human skin. Moreover, both MXenes and Ag/AgCl electrodes produced a similar ECG signal profile with comparable signal-to-noise ratio (SNR_MXene_ = 7.1, SNR_Ag/AgCl_ = 6.7). They further carried out other experiments to demonstrate the potential of their printed MXenes electrode for biosensor applications. In the first biosensor experiment, they attempted to detect Na^+^ cations in sweat. Na^+^ ion-sensitive membranes (ISM) were drop-casted on top of MXenes’ printed electrode, and the sensitivity of the Na^+^ was evaluated by recording its open circuit potential (OCP) vs. Ag/AgCl while varying concentration of Na^+^. They reported that the ISM/MXenes sensors’ response was linear to the concentration of the Na^+^ with a slope of 40 mV/decade. Their second MXenes biosensor experiment involved the integration of interferon gamma (IFNγ) antibody on MXenes electrode. Similar to the previous experiment, the IFNγ antibody functionalization on MXene’s electrode was done by drop-casting method. Physical absorption of the antibody allowed for binding with MXene’s surface; therefore, an immunosensor was successfully constructed and used for cytokine proteins detection in different concentrations. The sensitivity of the immunosensor was reported to be 3.9 mV per decade. Saleh’s study of MXenes as skin conformable electronics is considered to be one of the most important; however, it suffers from a lack of analysis in real samples.

Zhang and co-workers developed a voltametric sensor using screen-printed modified with MXenes for detection of AC and INZ [177]. A SPE surface was functionalized through drop-coating method of MXenes solution. Detection of INZ and AC in 0.1M H_2_SO_4_ supporting electrolyte on the MXenes/SPE gave a well-defined DPV current signal without overlapping with each other, thereby suggesting that this sensor was potentially used for simultaneous detection. The operating range of this sensor for AC detection was 0.25 to 2000 μM, while the range for INZ detection was 0.1 to 4.6 mM. Both detections demonstrated linear sensing responses proportionate to concentrations. The LODs of this sensor were 0.048 μM and 0.064 mM for AC and INZ detection, respectively. Moreover, these sensor performances were comparable to or even better than those of other works in the literature that used different sensing platforms. Zhang et al. concluded that their remarkable sensing performances of this sensor was attributed to MXenes material. Firstly, Ti_3_C_2_T_x_ MXenes is titanium-rich material. It not only provides robust carbon support but also allows for an effective conductive channel for electrons. Secondly, MXenes favors many active sites’ exposure and facilitates faster electron transfer during the electrocatalytic process owing to its accordion-like layered structure. Lastly, Ti_3_C_2_T_x_ MXenes is generally a negatively charged surface with -F, -OH and =O functional groups; therefore, it is beneficial to the aggregation of positively charged analytes such as INZ.

**Figure 11:**
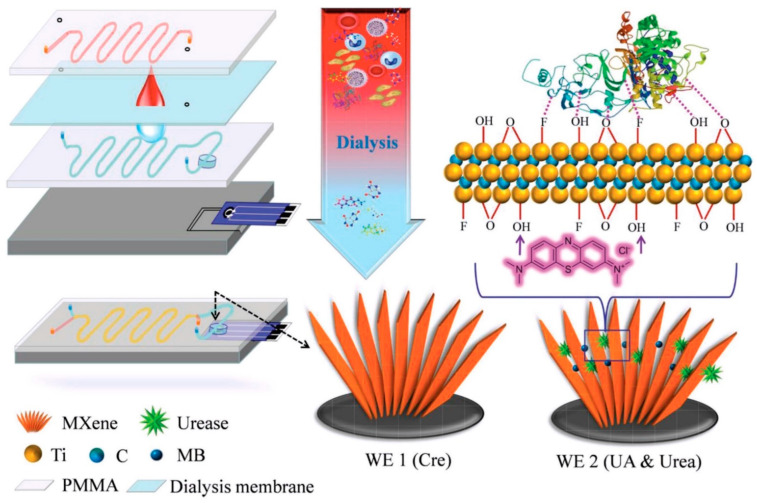
Structure of a microfluidic system with MXenes/MB/urea SPE for whole blood detection. Reprinted with permission from reference [178]. Copyright 2019 John Wiley and Sons.

Integration of an electrochemical sensor with a microfluidic system has certainly paved a new pathway for high sensitivity sensing with automation and miniaturization features. For example, Liu et al. implemented MXenes-modified SPE with a microfluidic system for a direct and continuous multicomponent analysis of whole blood. The microfluidic system was comprised of four layers, as shown in Figure 11, where the top layer had a channel for blood flow and the second layer, which was made up of dialysis membrane, allowed for small molecules to penetrate (size molecule < 1000 Da), while the third layer was designed with a flow channel for isotonic solution and a detection chamber. The analytes in blood were dialyzed into the channel at this layer and gathered in detection chamber. Lastly, at the very bottom layer, the electrochemical electrode was positioned for sensing purposes. For the sensing electrode part, the authors carefully designed the sensing matrices on SPE. To increase sensing surface area and enhance electrocatalytic activity, the SPE sensing surface was coated with MXenes, followed by the addition of methylene blue (MB) to adjust the background signal and aid measurement stability. Finally, the authors immobilized urease with the aid of glutaraldehyde on the MXene surface to generate a specific signal for urea analysis. This novel biosensing system demonstrated detection of creatinine (Cre) with LOD of 1.2 × 10^−6^ M in the linear range of 10–400 × 10^−6^ M. While the detection of uric acid (UA) gave current responses that were not so linear with the UA concentration, it is still acceptable as a general logarithmic trend. The logarithmic relation between current response and concentration was in the range of 30–500 × 10^−6^ M with LOD of 5 × 10^−6^ M. With the same sensing system, the authors performed the determination of urea. They reported that the electrical response displayed linear relation with urea concentration in the range of 0–3 × 10^−3^ M. The same sensing system exhibited excellent performance in the quantification of those tricomponents in real whole blood sample analysis. The recoveries of the novel biosensing system were 90.41–98.28% (UA), 90.16–99.82% (urea), and 83.59–95.66% (Cre).

## 6. Hexagonal Boron-Nitride (h-BN)

**Figure 12:**
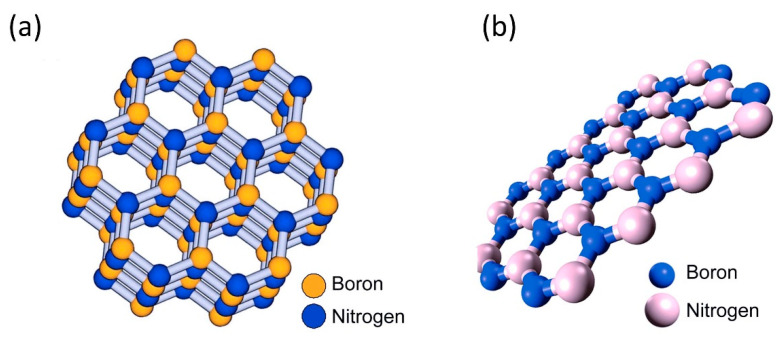
Illustration of the atomic structure of hexagonal boron-nitride (**a**) in a bulk structure, (**b**) in a monolayer structure. Reprinted from [179], with the permission of AIP Publishing.

h-BN is one of the emerging materials in the 2D material class with promising potential for electronics applications. The h-BN nanosheet has a dense honeycomb layered structure analogous to that of graphite, which contains sp^2^ hybridized B-N bonds. In a h-BN bulk structure, a few single h-BN layers are stacked together through Van der Waals interaction, which boron and nitrogen atoms are placed alternately as shown in Figure 12a, while in every single layer of h-BN, B and N are linked by a strong covalent bond in a sp^2^-hybridized honeycomb lattice (Figure 12b) [179]. Among the other 2D materials, hexagonal boron-nitride is an unpopular material for electronic applications due to initial reports of it being non-conductive [180,181]. Despite the fact that h-BN is reported as a non-conductive material, Ferrari et al. provide a comprehensive review of its use in electroanalytical sensing platforms [36]. Herein, we will specifically cover the topic of h-BN as material for sensing on printable electrode platform.

Yue et al. constructed a biosensor on SPE using h-BN conjugated with silver nanoparticles (AgNPs) as a probe for the detection of scopoletin [182]. Before being drop-cast on a SPE WE, AgNPs were first deposited onto an h-BN layer to form a nanocomposite. The AgNPs on hBN were found to increase the active site numbers, accelerate the electron transfer, and expand sensor surface area. Through CV analysis, the hBN/AgNPs/SPE sensor exhibited high electrocatalytic activity towards scopoletin, as its oxidation peak current increased significantly compared to scopoletin on bare SPE sensor. Further, the sensitivity of the modified SPE towards scopoletin was analyzed with DPV signals, which were recorded through scopoletin oxidation at concentrations ranging from 2 μM to 0.45 mM, yielding LOD of 0.89 μM with SNR of 3. The research group also reported success in the determination of scopoletin in *Atractylodes macrocephala* herb. The average concentration of scopoletin found in the herb was 5.478 μM. This value has been verified with HLPC Agilent 1100 to confirm the accuracy of the hBN/AgNPs/SPE sensor. A study by Öndes et al. proves that biological recognition element can be directly tethered on h-BN nanosheet for detection of ovarian cancer [183]. The surface electrode of SPE was modified with boron nitride nanosheets, followed with immobilization of anti CA125 antibody on the nanosheet for probing purposes. Previous research has shown that the BN surface has natural affinity for protein bindings, which the lysine unit of the protein has strong attraction towards the boron atom on the BN nanosheet [184]. Therefore, the protein can attach to BN nanosheets through absorption without the need for coupling agent. Öndes et al. recorded significant decreased in CV redox current when analyzed BN on SPE. This result is attributed to the insulator characteristic of BN, whose covalent layer of boron and nitrogen atom localizes free electrons. Interestingly, after immobilization of the antibody, the redox current magnitude increased. This is a rather unexpected result. This result contradicted other previous studies in which the immunosensor DPV readings decreased with increasing antigen concentration [185,186,187,188]. The researchers described the cause of the DPV’s decreased peak current with increasing antigen concentration as being due to electron transferability between the electrode surface and redox probe being hindered by antigen-antibody bindings. In corroboration with increasing redox current, the authors of the presented study presumed that the antibody molecules might have entered between BN layers, thus increasing the number of transfer electrons. Their hypothesis seems to be consistent with other research, which found that molecules and ions can be presented in the intra-layer spacing between the BN sheets [189]. Through DPV, the established immunosensor displayed 1.18 U/mL LOD using ferro/ferri cyanide as a redox probe. The immunosensor also showed good detection in a linear range of 5–100 U. To assess its practicality in real-world applications, the immunosensor was tested in artificial human serum spiked with 20 and 50 U/mL of CA125. The DPV measurement was carried out for this experiment, and they reported average relative recoveries of 98.13% and 101.22% for 20 and 50 U/mL CA125, respectively. Kokulnathan’s research team capitalized on the bismuth oxide and hexagonal boron nitride (Bi_2_O_3_/h-BN) nanocomposites heterostructure to construct a disposable electrochemical sensor for detection of flutamide, which is commonly used in the treatment of prostatic cancer [190]. They modified a carbon SPE with the Bi_2_O_3_/h-BN nanocomposite through dripping method, followed by drying process in an oven. The strong interaction between 2D hBN and bismuth oxide modifies the physical and electronic properties of the nanocomposite. The modified electrode was reported to have become rough, which gives it favorable characteristics for electrochemical sensing. Rough surfaces provide a larger surface area and thereby enhance sensing efficiency. The electrochemical detection of flutamide was established through the existence of free carriers in the nanocomposite heterojunction. The LOD value of 9.0 nM was achieved with good linear ranges of 0.04–87 μM obtained by the DPV response method.

## 7. Conclusions

The advantages of low cost and high reliability have drawn significant research attention to printed electrochemical sensors. They have long been developed with various types of electrode materials such as carbon, gold, platinum, and so forth that enable electrocatalytic reactions for high sensitivity sensors. We have surveyed recent advances in non-carbon 2D materials integration with printed electrochemical sensors and discussed key developments of the sensor from the perspective of materials. Within the past five years, TMDC’s MoS_2_ material has shown rapid advancement as a material for surface modification on the printed sensor. The popularity of MoS_2_ in printed electrochemical sensor applications stems from its versatile features. MoS_2_ makes a very ideal candidate for sensing material owing to its extraordinary electrical properties. Being physically almost similar to its counterpart graphene, but featuring tunable bandgap properties have certainly attracted much attentions to study MoS_2_ as a sensing material. Despite the fact that MoS_2_ materials have shown continuous advancement in the sensor field, TMDC materials are generally still in their infancy compared to long established graphene and silicon materials. Thus, it could be explained why the other TMDC materials (MoSe_2_, WS_2_, etc.) are still scarcely explored for printed electrochemical sensors. At a future time, we can anticipate many other TMDC compounds being capitalized as sensing materials. Similar to TMDC, MXenes is also a new 2D material that was introduced in 2011. MXenes have recently been gaining popularity as a preferred material for sensors owing to its high conductivity. However, MXenes require more time to establish in the sensor field as it is facing challenges at the synthesis level, such as easy aggregation and poor stability in oxygen atmosphere. In the case of hBN, it was the least popular as sensing material because of its insulator property. Having said that, hBN is really advantageous when combined with the other materials or elements as a sensing layer.

Let us now consider the challenges that are facing us in this printed sensor field. Despite the impressive achievements that have been made in this field, printed sensors are still having difficulties in a few areas and crucially need to be addressed. (1) To fabricate a very robust and sensitive sensing platform with low a detection limit and a wide dynamic range for application in real sample analysis still remain as the primary challenge in the sensors field. The complex matrices in real samples (e.g., urine, blood, human serum, lake water) will cause serious interferences, and the lower concentration of the target analytes will result in difficulty for direct determination. Due to competition between the target analytes and other molecules in the matrices to form bonds with the recognition sites on the sensing surface area, the sensor’s sensitivity is possibly lower in real samples. Furthermore, the interference in the matrices may also lead to false positive results. Choosing high performance electrode and sensing element materials is prominent to achieve high sensitivity sensor response, especially in complex matrices. Sensor integration with microfluidic systems that are equipped with filtering features can also help to filter out unwanted molecules, resulting in a more sensitive response. (2) Most of the studies reviewed in this article employed drop-casting as their primary method for sensor surface functionalization and modification. This technique, however, has raised concerns about the robustness and reproducibility of sensors. In reality, drop-casting for surface functionalization is not a very straightforward technique. The execution of the technique is very simple; however, it involves a complex process that is controlled by particle interactions, molecular packing constraints, together with kinetic and thermodynamic factors. In addition, as we discussed in the introduction part of this article, the drop-cast technique has the tendency to produce a coffee ring effect, where modifier particles are not evenly distributed on the sensing surface. Moreover, due to the sparse coverage, it cannot be guaranteed that all the modifier particles are in electrical contact with the substrate or are not aggregated. Consequently, these two factors can affect the signal reproducibility of the sensor. For real sample analysis, it is important to have a batch fabrication of sensors that will permit similar and consistent results. Therefore, future research might focus on the incorporation of modifier material in the electrode ink to fabricate the printed sensor. The major advantages of this method are that the sensor’s reproducibility is possible owing to evenly distributed recognition sites on the sensor surface, and the sensor signal can be improved. (3) In the healthcare field, flexible printed electrochemical sensors are usually associated with wearable monitoring systems. Despite the massive progress that has been made in wearable electrochemical sensor systems, the integration of power sources and the ability to communicate via wireless network remains a great challenge. Further research on the development of a power source that can scavenge power from the body via biokinetics or body heat is vital to enabling the application of printed electrochemical sensors as real-time wearable monitoring devices.

## Figures and Tables

**Table 1:** Performance of MoS_2_ ink towards dopamine sensing in the work of Pavličková et al. [102].

MoS_2_ Ink Sample	MoS_2_ Particle Size	Volume (wt%)	LOD	R^2^
Mo6-45	6 μM	45	246 nM	0.996
Mo6-25	6 μM	25	686 nM	0.995
Mo6-60	6 μM	60	669 nM	0.975
Mo90-25	90 nM	25	456 nM	0.982
Mo90-45	90 nM	45	865 nM	0.976

**Table 2:** MoS_2_ material applications on printed electrochemical sensor.

Electrode Structure	Target	SPE Modification Technique	Analysis Method	Linear Range	Detection Limit	Ref.
HM-Al^3+^-(2D-MoS2)/SPGrEs	Hydrazine	Drop-casting	Double pulse chronoamperometry	0–0.400 mM	1.05 μM	[136]
PANI/MoS_2_/SPE	Anti-cyclic citrullinated peptide (aCCP) antibodies	Drop-casting (MoS_2_) and electrochemically polymerization (PANI)	SWV	0.25 and 1500.0 IU/mL	0.16 IU/mL (PBS) and 0.22 IU/mL (10% Human Serum)	[137]
MoS_2_/PPyNPs/SPE	Ampicillin	Drop-casting	Amperometric	50–250 pg/L	10 pg/L (0.28 pM).	[138]
PMO-NiO/MoS2/SPCE	Cholesterol	Drop-casting and MO-polymerization	DPV	1 to 15 mg/dL	0.24 mg/dL	[139]
MoS_2_/CA/SPE	Troponin I	Electrospinning	EIS	10 fM to 1 nM	10 fM	[140]
RGO/MoS_2_/SPE	Cd^2+^, Hg^2+^, Pb^2+^ in rice	Drop-casting	DPV	5 to 160 μM (Cd^2+^) 5 to 160 μM (Hg^2+^) 10 to 3000 μM (Pb^2+^)	49.83 μM (Cd^2+^) 36.94 μM (Hg^2+^) and 733.90 μM (Pb^2+^)	[141]
CA-MoS_2_/SPE	Acute Myocardial Infraction (AMI)	Drop-casting	EIS	10 fM to 1 nM	10 fM	[142]
AChE@CHIT/TiO_2_/ MoS_2_/SPE	Monocrotophos pesticide	Electrodeposition and drop-casting	DPV	50 pM to 10 nM	50 pM	[143]
Probe-SH/MoS_2_/SPCE	Listeria or SARS-CoV-2	Drop-casting	DPV	NR	67.0 fM (Listeria) and 1.01 fM (SARS-CoV-2)	[144]
Co@MoS_2_/rGO/SPE	Glucose	Drop-casting	Amperometric	0 to 0.8 mM	30 nM	[145]
SPE-CB/MoS_2_	Cocoa catechins	Drop-casting	CV and DPV	0.12 to 0.25 μM	0.17 μM	[146]

NR: Not reported, MoS_2_: molybdenum disulfide, SPE: screen printed electrode, SPCE: screen printed carbon electrode, SPE-CB: carbon black SPE, SPGrEs: screen printed graphene electrodes, SWV: square-wave voltammetry, DPV: differential pulse voltammetry, EIS: electrochemical impedance spectroscopy, CV: cyclic voltammetry, HM-Al^3+^: hematin-aluminum complex, PANI: polyaniline, PPyNPs: PPyNPs: polypyrrole nanoparticles, PMO: poly (methyl orange), NiO: nickel oxide, CA: cellulose-acetate, AChE: acetylcholinesterase, CHIT: chitosan, TiO_2_: titanium oxide, Probe-SH: thiolated synthetic DNA sequence probe, Co: cobalt, rGO: reduced graphene oxide.

## Data Availability

Not applicable.
